# Rare case of simultaneous occurrence of chronic neutrophil leukemia and T lymphoblastic lymphoma: case report and literature review

**DOI:** 10.1007/s00277-024-05759-z

**Published:** 2024-08-06

**Authors:** Shi-xuan Wang, Fang Wang, Ye-chao Tu, Yu-lan Zhou, Song-tao Tu, Jie-yu Wang, Ke-bing Lv, Fei Li

**Affiliations:** 1 Center of Hematology, The First Affiliated Hospital of Nanchang University, Nanchang, China; 2 Jiangxi Clinical Research Center for Hematologic Disease, Nanchang, China; 3 Institute of Lymphoma and Myeloma, Nanchang University, Nanchang, China

**Keywords:** Chronic neutrophil leukemia, T-lymphoblastic leukemia, Selinexor, EP300, SMARCA4

## Abstract

Chronic neutrophil leukemia (CNL) is a rare and life-threatening disease. Cases of CNL combined with lymphoma are rare. Here, we report a case of CNL with T-acute lymphoblastic leukemia/lymphoma (T-ALL/LBL) in a 28-year-old male. After a regimen of ruxolitinib, VICLP (Vincristine, Idarubicin, Cyclophosphamide, Prednisone, Peg-asparaginase) regimen, high-dose cytarabine, and methotrexate regimens, the patient’s bone marrow condition partially resolved. However, when the disease relapsed four months later, despite attempts with selinexor, venetoclax, and CAG(aclarubicin hydrochloride, Algocytidine, Granulocyte Stimulating Factor) chemotherapy, the leukocytes and peripheral blood primitive cells reduced, but the bone marrow did not achieve remission. This pathogenesis may be related to microenvironmental immune escape under prolonged inflammatory stimulation and gene disruption affecting protein function due to colony-stimulating factor 3 receptor gene (CSF3R) mutations. For this type of disease, early intervention may delay disease progression.

## Introduction

Chronic neutrophil leukemia (CNL) is a rare BCR/ABL-negative myeloproliferative neoplasm (MPN) whose molecular biology is based on mutations in the CSF3R (Colony Stimulating Factor 3 Receptor). CNL exhibits substantial clinical and prognostic heterogeneity. Conversely, T-ALL/LBL is a highly aggressive malignancy that originates from immature T lymphocytes. Its diagnosis relies on immunophenotypic evidence of the T-cell lineage, with CD3 being the most specific T-cell marker. Here, we report the second case of simultaneous occurrence of CNL and T-ALL/LBL.

## Case description

**Fig. 1:**
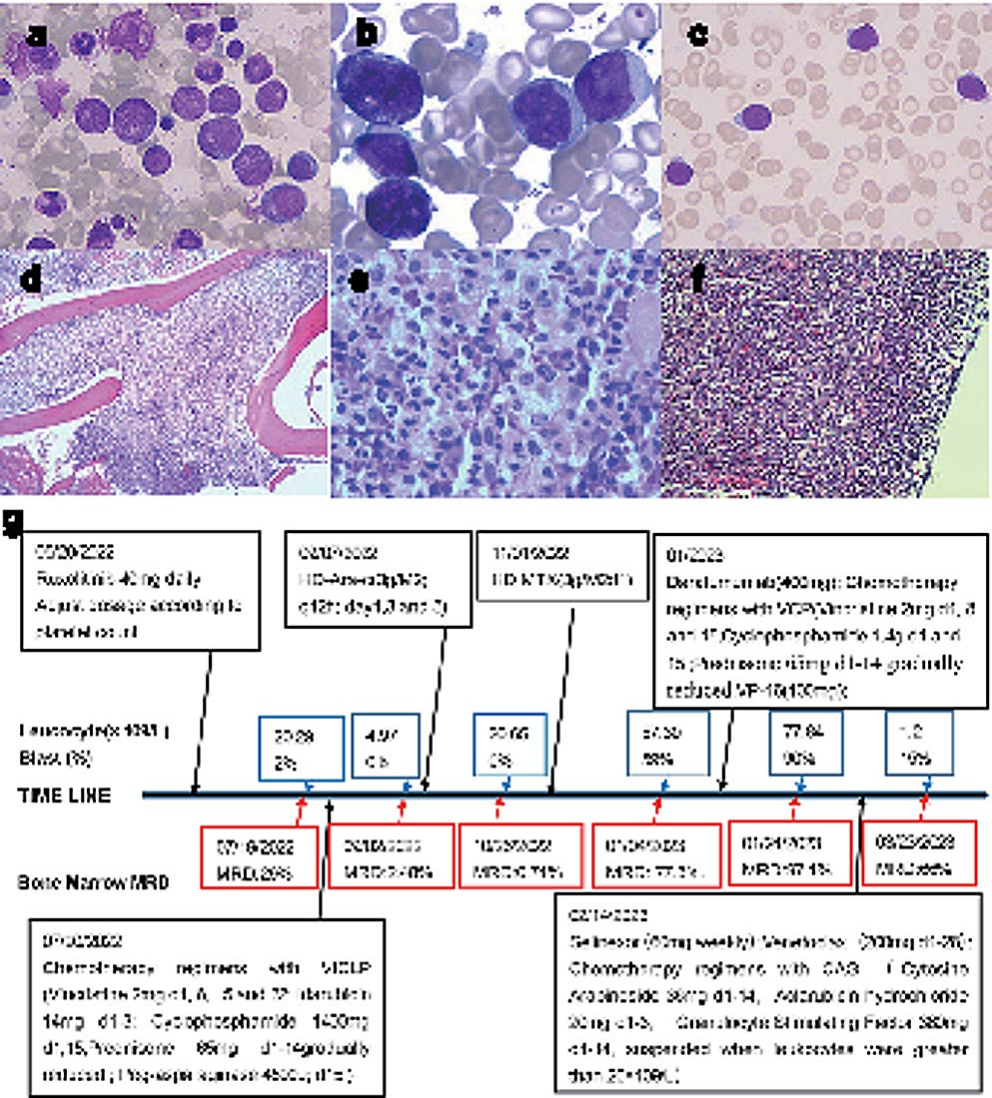
Bone marrow morphology: **a.** Bone marrow morphology: a. On November 2, 2020, markedly hypercellular granulopoiesis without an increase in blast cells **b**. On May 18, 2022, myeloid cells were actively proliferating. Increased and thickened granulocyte cytoplasmic granules and bone marrow primitive cells were seen **c**. On July 19, 2022, lymphocyte proliferation was active, and many blast cells and lymphoblast were seen. (Wright–Giemsa stain, 1000×). Bone marrow biopsy showed markedly myeloid hyperplasia with focal fibrous tissue hyperplasia and reticular fiber staining (MF-2, focal) **(d)** (10 × 10) and **(e)** (10 **×** 40), (HE and PAS staining). **(f)** Lymph node biopsy conducted on May 5, 2022 (HE Staining, 10 × 40). **(g)** The disease treatment timeline of the patient. HD-MTX: High-Dose Methotrexate ; HD-Ara-c : High-Dose Cytosine Arabinoside

A 28-year-old Chinese male with splenomegaly and polyarticular pain presented to our rheumatology department in October 2020. SPECT detected abnormalities in bone metabolism. The platelet count was 98 × 109 /L, hemoglobin (Hb) 9.1 g/dL, and the white blood cell (WBC) count was 25.50 × 109 /L (neutrophils, 91.6%). Bone marrow (BM) evaluation revealed active BM proliferation with 72.5% neutrophils (Fig. 1a**)**, and primitive immature cells were visible. Furthermore, the BM immunophenotyping revealed that granulocytes comprised 82.96% of the sample and expressed (approximately 80%) CD33, CD13, CD10, and CD15 with an abnormal expression curve, indicating an abnormal development of granulocytes. Reticular fiber staining showed MF-1. The patient was initially diagnosed with psoriatic arthritis and treated with diclofenac sodium and methotrexate, which improved joint pain and rash. The fractional abundance (FA) of CSF3R p.T6181 and CSF3R p.Y787* mutations by ddPCR for the genomic DNA obtained from the BM was 44.10% and 49.40%, respectively. The patient was negative for mutations in SETBP1 and ASXL1. Because the patient did not meet the criteria for other MPN disorders, CNL was diagnosed according to the WHO classification criteria, classified as High-risk CNL based on the operational risk model predicting long-term survival in CNL, which was developed from data captured from 19 molecularly annotated, patients with CSF3R-mutated CNL from the Mayo Clinic [1]. However, the patient refused to use Ruxolitinib.

The patient was hospitalized in May 2022 with multiple bilateral neck masses. BM smear showed active BM proliferation with 8.5% primitive cells (Fig. 1b). A subsequent BM biopsy (Fig. 1d, e) showed active proliferation of BM cells (> 90%) with an increased percentage of granulocytes and immature myeloid cells. Moreover, there was focal fibrous tissue hyperplasia(MF-2). Additional immunophenotyping of BM cells demonstrated that 5.31% of the nuclear cells were abnormal T lymphoblasts, with a significant increase in the proportion of granulocytes and a decrease in erythroid cells. The abnormal cell population strongly expressed (approximately80%) CD7, CD34, CD33, CD13, CD123, CD99, and CD10 with partial expression (approximately40%) of TDT, CD19, and cCD3(>10%). Weak expression (approximately 20%) of CD38, HLA-DR, CD11b, and CD5 was also observed, whereas no expression of CD117, CD4, CD2, CD8, CD1a, cCD79a, MPO, or other myeloid or lymphoid markers was observed. Overall, these findings were consistent with the phenotype of T lymphoblastic cells. The lymph node (LN) biopsy (Fig. 1f) showed a large area of diffuse infiltration of medium-sized atypical lymphocytes into the dermis and subcutaneous tissue. Immunohistochemically, the neoplastic lymphoid cells were positive for CD10, CD7, CD43, CD3 (partial), PAX-5 (partial abnormal), and CD99 and Ki67(90%). The karyotype is normal(46, XY [20]). The BCR-ABL fusion gene was not detected. Based on these, the patient was diagnosed with CNL combined with T-LBL. The NGS of LNs showed mutations in EP300 exon 3 and SMARCA4 exon 14. After treatment with ruxolitinib for 20 days, the patient’s spleen and LNs decreased in size. The patient refused further chemotherapy and treatment with hematopoietic stem cell transplantation (HSCT) and then continued treatment with ruxolitinib.

In July 2022,the patient had an enlargement of the neck mass.BM smear (**Fig. 1c**) showed active lymphocyte proliferation with 52.5% lymphocytes, of which 42.5%were lymphoblasts and prolymphocytes. Using a multicolor flow cytometry (MFC) minimal residual disease (MRD) assay with a sensitivity of 10 − 4,and 29.03% of the abnormal cell(CD7 + CD34 + sCD3-CD10+/-CD45±) populations were detected. The disease had progressed to ALL. Successively, he received remission induction with the VICLP regimen and consolidation chemotherapy with the High-Dose Cytosine Arabinoside (HD Ara-c) and High-Dose Methotrexate (HD-MTX) regimens (Fig. 1g). Efficacy was assessed as a partial response. After four months, he developed a new lump in his right elbow. A BM smear revealed 67.0% primitive and immature lymphocytes. Pathological biopsy of the mass suggested a diffuse patchy distribution of lymphocytes with infiltrative growth. Furthermore, immunohistochemistry suggested the neoplastic lymphoid cells had strong expression(approximately 80%) for CD3, CD43, CD10, CD5, TdT and CD38, Ki67 (60%), and weak expression (approximately 20%)of CD99, CD79a, and Bcl-2. Subsequently, chemotherapy regimens such as VCP (vincristine, cyclophosphamide, prednisone) regimen, daratumumab (400 mg weekly, 3 weeks), and etoposide were administered, but he had poor tumor control. Subsequently, he was treated with Selinexor (60 mg weekly), venetoclax (200 mg Day1-28), CAG chemotherapy from February 14, 2023. The patient’s elbow mass and spleen size reduced after this chemotherapy regimen, and the white blood cells and peripheral blood primitive cells (Fig. 1g) were reduced. Unfortunately, his BM has not achieved remission. Finally, he was discharged from the hospital. Three months later, we followed up with his family by phone and were told he had died.

## Result and discussion

Most CNL patients carrying CSF3R mutations were first identified in 2013 by Maxson et al. [2]. Then, Pardanani et al. demonstrated the presence of CSF3R T618 I mutation in 83% of WHO-defined CNL patients [3]. Besides two types of CSF3R mutations, genes involved in epigenetic modifications, spliceosomes, and signaling pathways often occur in CNL and play a role in its occurrence and development. In the largest cohort of 39 CNL cases, the most commonly affected gene in CNL was ASXL1, accounting for approximately 77% of patients with frameshift or nonsense mutations. TET2, SRSF2, U2AF1, and SETBP1 mutations were present in approximately 21%, 44%,15%, and 41% of cases, respectively [4]. Multiple mutations (> 3) were strongly associated with the development of leukemic transformation and shortened survival. BM reticulin stain in CNL is usually normal; in a few cases, it may show minimal fibrosis but should not exceed a grade of 1+. However, CNL is a special type of MPN in which myelofibrosis may occur as the disease progresses. Our literature review revealed that in 2018, Wu XB et al. reported a case of CNL CSF3R mutation with myelofibrosis (positive Gomori staining (++++~++++)) and noted that the presence of myelofibrosis in CNL suggests that the disease was evolving to the end-stage [5]. BM pathology in our patient 17 months after the diagnosis of CNL revealed focal MF-2 staining of BM reticular fibers, suggesting that increased BM fibrosis may be a marker of disease progression or evolution.

The case of CNL combined with lymphoma is very rare. Only one case of CNL combined with B-LBL was reported by Joseph Aoki on Blood Advance in 2020. Therefore, the relationship between them remains unclear. In addition, an Italian study suggested that the risk of patients with MPN developing lymphoid neoplasm was 2.79-fold higher (95% confidence interval (CI): 1.80–4.33 ) than in the general population [6].

The reciprocal pathogenic relationship between MPN and lymphoma is currently unclear. Several studies have suggested that lymphoma cells and MPN may originate from a common lymphoid-myeloid progenitor cell, leading to the sequential development of both diseases [7, 8]. Some studies have suggested that ruxolitinib treatment may be associated with an increased risk of developing lymphoma in MPN patients with JAK2V617F mutation [9–11]. Since treatment with JAK inhibitors may be associated with an increased risk for lymphoma. The professional suggestion of Elisa Rumi et al. is to detect the presence of a B-cell clone before starting ruxolitinib to balance the risk-benefit ratio [12]. In 2020, Joseph Aoki reported a confirmed case of CNL that transformed into B-LBL, mentioning that the combination of mutations in CSF3R, ASXL1, SRSF2, and RUNX1 may be critical in driving B-cell leukemogenesis; however, no definitive study has been conducted [13]. We report this case involved two CSF3R mutations (p.T6181 and P.Y787*), EP300 p. H2339Y mutation and SMARCA4 p. R704Q mutation. Whether there is an association among these gene mutations is unclear and is presumably involved in the development of lymphoma.

The current treatment strategy for CNL is similar to that of other MPNs. Traditional drug therapies are less effective. The prognosis is extremely poor when CNL progresses to an accelerated or acute transformation phase. Treatment in the acute transformation phase is usually based on an AML induction remission regimen. However, this approach has a low complete remission rate. In recent years, the clinical application of molecularly targeted drugs in CNL has confirmed that patients with CSF3R T618I mutations are sensitive to the JAK2 inhibitor, ruxolitinib. Patients carrying the CSF3R S783fs truncation are sensitive to dasatinib treatment. Despite partial progress in targeted therapy, the overall prognosis is poor, with a short median overall survival. Patients with the CSF3R T618I mutation had a significantly shorter median survival time than those with the other CSF3R mutation. HSCT is still considered the only possible cure for this disease and should be the first choice for young patients.

Since CNL combined with T-LBL is rare, only reported cases of MPN combined with lymphoma are available to understand the relevant treatment, efficacy, and prognosis. The reported cases of MPN combined with lymphoma were mainly treated with hydroxyurea and JAK inhibitors to alleviate the symptoms of MPN and delay disease progression and with relevant chemotherapy regimens for lymphoma to achieve remission. The prognosis of the coexisting malignancies remains unclear because there are few observable cases, and the follow-up of patients with reported cases is incomplete. As illustrated in this case, the patient’s NGS revealed the presence of a compound mutation in CSF3R p.T6181 and CSF3R p.Y787*, which can promote neutrophil proliferation by activating the downstream MAPK pathway. Given that the CSF3R T6181 mutation was sensitive to ruxolitinib treatment, the patient was recommended to take oral ruxolitinib as early as possible, but the patient refused. Later, the patient was diagnosed with CNL complicated by T-LBL and was not treated with chemotherapy. Although oral ruxolitinib resulted in a reduction in spleen size, the disease quickly progressed to ALL. The delayed diagnosis of CNL in this patient may have led to clonal evolution and the development of other hematologic tumors, suggesting that early treatment with ruxolitinib may have achieved better outcomes. Maxson et al. found that the complex mutations were characterized by both truncating mutations and membrane proximal mutations, and in vitro experiments showed that the complex mutants possessed strong cell proliferation ability; it was hypothesized that they might be more oncogenic than truncating mutations or membrane proximal mutations [14] Mohammad Azam et al. suggested that compound mutations in the CSF3R gene induce aggressive lethal leukemia in mice that show resistance to both JAK and SRC inhibitors in vitro and are more sensitive to trametinib, an inhibitor of the MAPK signaling pathway [15]. Also, some data suggest that Mek1/2 inhibition alone could be effective against CSF3R-expressing cells, while a combination of Mek1/2 and Jak2 inhibitors would perhaps be more effective in targeting the ruxolitinib-resistant compound mutations [15]. Therefore, the compound mutation of this patient may be the reason for the poor efficacy of ruxolitinib. Although remission of BM morphology was achieved with the VICLP, HD-Ara-c, and HD-MTX regimens, the patient did not undergo allogeneic HSCT for personal reasons, after which the disease rapidly relapsed. Despite aggressive administration of CD38 monoclonal antibody combined with chemotherapy regimen and attempted treatment with BCL-2 and exportin-1 inhibitors in combination with CAG regimen, the patient did not achieve a therapeutic response.

In conclusion, we report a case that rapidly progressed to ALL after a diagnosis of CNL combined with T-LBL. Presently, reports of CNL combined with T-LBL are rare, and their clonal origin remains unclear. Future studies, such as molecular and cytogenetic studies that explore their relationship would be intriguing.

## Data Availability

No datasets were generated or analysed during the current study.
